# Patterns of individual non-treatment during multiple rounds of mass drug administration for control of soil-transmitted helminths in the TUMIKIA trial, Kenya: a secondary longitudinal analysis

**DOI:** 10.1016/S2214-109X(20)30344-2

**Published:** 2020-10-15

**Authors:** William E Oswald, Stella Kepha, Katherine E Halliday, Carlos Mcharo, Th'uva Safari, Stefan Witek-McManus, Robert J Hardwick, Elizabeth Allen, Sultani H Matendechero, Simon J Brooker, Sammy M Njenga, Charles S Mwandawiro, Roy M Anderson, Rachel L Pullan

**Affiliations:** a Department of Disease Control, Faculty of Infectious and Tropical Diseases, London School of Hygiene & Tropical Medicine, London, UK; b Department of Medical Statistics, Faculty of Epidemiology and Population Health, London School of Hygiene & Tropical Medicine, London, UK; c Eastern and Southern Africa Centre of International Parasite Control, Kenya Medical Research Institute, Nairobi, Kenya; d Pwani University Bioscience Research Centre, Pwani University, Kilifi, Kenya; e London Centre for Neglected Tropical Disease Research, Faculty of Medicine, Department of Infectious Disease Epidemiology, School of Public Health, St Mary's Campus, Imperial College London, London, UK; f Neglected Tropical Diseases Unit, Division of Communicable Disease Prevention and Control, Ministry of Health, Nairobi, Kenya

## Abstract

**Background:**

Few studies have been done of patterns of treatment during mass drug administration (MDA) to control neglected tropical diseases. We used routinely collected individual-level treatment records that had been collated for the Tuangamize Minyoo Kenya Imarisha Afya (Swahili for Eradicate Worms in Kenya for Better Health [TUMIKIA]) trial, done in coastal Kenya from 2015 to 2017. In this analysis we estimate the extent of and factors associated with the same individuals not being treated over multiple rounds of MDA, which we term systematic non-treatment.

**Methods:**

We linked the baseline population of the TUMIKIA trial randomly assigned to receive biannual community-wide MDA for soil-transmitted helminthiasis to longitudinal records on receipt of treatment in any of the four treatment rounds of the study. We fitted logistic regression models to estimate the association of non-treatment in a given round with non-treatment in the previous round, controlling for identified predictors of non-treatment. We also used multinomial logistic regression to identify factors associated with part or no treatment versus complete treatment.

**Findings:**

36 327 participants were included in our analysis: 16 236 children aged 2–14 years and 20 091 adults aged 15 years or older. The odds of having no treatment recorded was higher if a participant was not treated during the previous round of MDA (adjusted odds ratio [OR] 3·60, 95% CI 3·08–4·20 for children and 5·58, 5·01–6·21 for adults). For children, school attendance and rural residence reduced the odds of receiving part or no treatment, whereas odds were increased by least poor socioeconomic status and living in an urban or periurban household. Women had higher odds than men of receiving part or no treatment. However, when those with pregnancy or childbirth in the previous 2 weeks were excluded, women became more likely to receive complete treatment. Adults aged 20–25 years were the age group with the highest odds of receiving part (OR 1·41, 95% CI 1·22–1·63) or no treatment (OR 1·81, 95% CI 1·53–2·14).

**Interpretation:**

Non-treatment was associated with specific sociodemographic groups and characteristics and did not occcur at random. This finding has important implications for MDA programme effectiveness, the relevance of which will intensify as disease prevalence decreases and infections become increasingly clustered.

**Funding:**

Bill & Melinda Gates Foundation, Joint Global Health Trials Scheme of the Medical Research Council, UK Department for International Development, Wellcome Trust, Children's Investment Fund Foundation, and London Centre for Neglected Tropical Diseases.

## Introduction

Control of the five neglected tropical diseases with the highest burden—schistosomiasis, lymphatic filariasis, onchocerciasis, trachoma, and infection with soil-transmitted helminths—involves mass drug administration (MDA), in which an entire eligible population or the most vulnerable subgroups receive treatment without diagnosis.^1^ The success of MDA depends on the percentage of the target population who take treatment.^2^ Mathematical modelling has highlighted the potential for systematic non-treatment, where the same individuals are not treated over multiple rounds of MDA, to drive persistence of infection reservoirs in the target population.^3^, ^4^, ^5^ Studies have investigated determinants of reach and uptake within preventive MDA programmes, but few have quantified receipt of treatment over time in individuals to measure the extent of systematic non-treatment.^6^, ^7^, ^8^, ^9^ As with other neglected tropical diseases for which preventive chemotherapy is appropriate, guidance on optimising treatment for soil-transmitted helminths will need to be based on understanding of patterns and factors associated with non-treatment during MDA.^10^, ^11^

The Tuangamize Minyoo Kenya Imarisha Afya (Swahili for Eradicate Worms in Kenya for Better Health [TUMIKIA]) trial was a 2-year community-based, cluster-randomised controlled trial done between 2015 and 2017 in Kwale county, Kenya, to evaluate the effectiveness and cost-effectiveness of school-based versus community-wide deworming on transmission of soil-transmitted helminths.^12^ Located on the south coast, Kwale is predominantly rural, is among the poorest counties, and has poor access to water and sanitation.^12^ Annual school-based deworming has been implemented in Kwale as part of the national programme since 2012, and community-based MDA for lymphatic filariasis has been implemented by the Kwale county Ministry of Health intermittently since 2003.^13^ The trial demonstrated that community-wide deworming achieved high coverage across all key demographic groups and was more effective than annual school-based deworming at reducing prevalence and intensity of hookworm infection.^14^ In this analysis, we use routinely collected data on treatment across the four treatment rounds in the TUMIKIA trial to assess patterns of non-treatment and identify subgroups of the population at risk of systematic non-treatment.

**Research in context**

**Evidence before this study**

Control efforts for many neglected tropical diseases, including soil-transmitted helminths, schistosomiasis, lymphatic filariasis, onchocerciasis, and trachoma, focus on mass drug distribution (MDA), which is treatment of an entire eligible population or most vulnerable subgroups without diagnosis. The effectiveness of this approach for controlling morbidity or reducing transmission depends upon the percentage of the population treated in each round. Mathematical modelling has highlighted that persistent non-treatment of individuals across multiple MDA rounds could negatively affect interruption of transmission of soil-transmitted helminths. An earlier systematic review, on which we have based this study, also highlighted that few studies have quantified receipt of treatment over time. Improved understanding of patterns and factors associated with non-treatment across MDA rounds will benefit efforts to strengthen delivery and optimise treatment for soil-transmitted helminths and other neglected tropical diseases.

**Added value of this study**

This analysis was done after linking the baseline population of the biannual MDA group in the TUMIKIA trial in Kwale County, Kenya, to longitudinal records of receipt of treatment in the four treatment rounds of the study. Among children (age 2–14 years) and adults (age ≥15 years) we found that the same individuals repeatedly missed treatment. Although the proportion of participants who received no treatment in all four rounds was low, more than half of individuals received fewer than four treatments. For children, school attendance and rural residence were protective against receiving part or no treatment. Among adults, those aged 20–25 years were most likely to receive part or no treatment, and women were less likely than men to receive all four treatments.

**Implications of all the available evidence**

Very few studies have quantitatively examined patterns of treatment across multiple rounds of MDA for control of neglected tropical diseases. Our results indicate distinct demographic subgroups that did not complete treatment. Further research is needed to examine how the extent of non-treatment might jeopardise MDA outcomes and how to strengthen MDA implementation approaches to increase coverage.

## Methods

### Study design and participants

The TUMIKIA trial design, baseline findings, and effects have been described previously.^12^, ^13^, ^14^ Briefly, 120 community units (clusters) of approximately 1000 households were randomly assigned to receive albendazole through annual school-based treatment targeting children aged 2–14 years or through annual or biannual community-wide treatment targeting all ages. The primary outcome was community hookworm prevalence, which was assessed at baseline and at 12 and 24 months through cross-sectional surveys. The trial protocol was approved by the Kenya Medical Research Institute and National Ethics Review Committee (SSC number 2826) and the London School of Hygiene & Tropical Medicine Ethics Committee, London, UK (7177). Written informed consent was sought from the household head or adult answering household-level questionnaires.

In the present study, participants in the baseline survey who were randomly assigned to receive biannual treatment were linked to their treatment records from the four rounds of MDA during the trial. We included individuals recorded to be aged 2 years or older at baseline and not recorded as deceased or migrated in any round.

### Procedures

Households were enumerated at the start of the trial from listings provided by community health services and village leaders or from other community-based programmes. For each survey, 225 households per cluster were randomly selected from the enumeration listing. In consenting households, members were counted and their sociodemographic information and school attendance (for children) were recorded. Information was also collected on household assets, sanitation, hygiene, and water conditions. All data and GPS coordinates were collected on smartphones running the Android operating system and using SurveyCTO software (Dobility, Cambridge, MA, USA). Household locations were classified as urban, periurban, or rural, based on the 2015 World-Pop estimates of population density. Household remoteness was calculated as the Euclidean distance from a major road, using Open Street Map data. Location classification, remoteness calculation, and point-based extraction were done in ArcGIS version 10.3.^13^ Households without GPS coordinates were given the village mode or mean value for categorical and continuous measures, respectively.

Four rounds of treatment were provided to the biannual group over 2 years. Before each round of treatment, community mobilisation activities took place that included forums with chiefs, ward administrators, and community health assistants, and meetings held by and door-to-door visits made by village elders.^15^ Participants received directly observed treatment with 400 mg albendazole provided through the WHO donation programme to the Government of Kenya by GlaxoSmithKline (London, UK). Rounds one and three were provided in Jul 20–30, 2015, and May 26–June 3, 2016, respectively. Children aged 2–14 years at school received treatment as part of the National School-Based Deworming Programme (round 1, June 4, 2015; round 2, May 26, 2016), and all other eligible recipients were treated by community health volunteers in community-wide house-to-house visits organised by the Kwale county Ministry of Health. Rounds two and four were provided in Nov 23–30, 2015, and Oct 28–Nov 4, 2016, and all people were treated during house-to-house visits. In these rounds, individuals also received 6 mg/kg diethylcarbamazine citrate as part of the restarted national lymphatic filariasis elimination programme.^16^ Community health volunteers received 1 day of training on the delivery of treatment, and each was responsible for treating approximately 120 households over 8 days per round. Trial staff provided training and technical support but were not involved in the delivery of treatment to household members.

Community health volunteers were provided with household enumeration listings for their area, which included the name of the head of each household and the TUMIKIA trial house identification number. On paper registers for each household, community health volunteers recorded the name, cluster, TUMIKIA trial house identification number, date of first visit, and number of revisits. They also listed the names, sexes, ages, and school enrolment status of all people who had been living in the house in the previous 3 months. Next to each individual listing, community health volunteers recorded whether albendazole was swallowed at the first visit or a revisit, along with the date of treatment and any side-effects experienced. If albendazole was not swallowed, this was classified as non-treatment and the reason was recorded from the following list: temporarily absent; pregnant; having given birth within 2 weeks; refused; younger than 2 years; spat drug out; sick; migrated; died; or other. Participants recorded as recently dewormed (within the previous 8 weeks) in a school programme or at a health facility were recorded as having received treatment.

The paper treatment registers were collected and digitised with SurveyCTO following each community-wide treatment round. In rounds one, two, and three, only age and sex were digitised to estimate coverage by population group, but individuals' names were not digitised. Names were included in the round-three digitised registers. This dataset was used to prepopulate an electronic data entry form for the digitisation and linkage of paper treatment registers from round four. Information from earlier rounds were retrospectively added. Data officers confirmed that the households in the paper and digitised records matched based on the names of household members. They then matched each individual's name, age, and sex in in the paper register and digitised dataset. Following linkage of data for all four treatment rounds, another electronic data entry form was used to link households and individuals enumerated in the baseline cross-sectional household and parasitology survey to their treatment data.

### Outcomes

An individual was classified as being treated if they were recorded as having been directly observed to swallow albendazole at home or reported to have received it in a school or health facility within the previous 8 weeks. Among those without recorded treatment, reasons for non-treatment were reported in the registers for only approximately 10% of children and 20% of adults. To address these missing data, within rounds we combined people with recorded reasons for non-treatment and those who had no record available for that round, and used the indicator non-treatment. Frequencies of recorded reasons for non-treatment and absence of records are provided in the appendix (p 2). An individual's treatment status was summarised as complete if treatment was recorded in all four rounds, part if treatment was recorded in fewer than four rounds, and none if no treatment was recorded in any round.

### Statistical analysis

Separate analyses were done for children aged 2–14 years, and adults (age ≥15 years) of associations between non-treatment in round one and other factors; between non-treatment in a given round and non-treatment in the previous round; and between factors and part or no treatment. In the TUMIKIA trial, 40 clusters were randomised to each treatment group, and 225 households per cluster were assessed in each prevalence survey, which was estimated would provide 80% power to detect 8% difference in prevalence between groups with a 2·5% significance level.^12^ The sample size for this study was determined by the number of households in the baseline survey that were randomly assigned to the biannual treatment group.

We examined frequencies of non-treatment within and across rounds using histograms generated with the UpSetR package in R version 3.5.0.^17^ We estimated associations between non-treatment in the first round and the following candidate predictor variables at baseline: age group; sex; school attendance by children; household socioeconomic status (least poor, poor, or poorest) derived from a factor analysis of assets; large household size (more than six members); remote household (>4 km from major road); and household location (urban and periurban *vs* rural). An additional predictor for children was based on whether the head of household was not treated in a given round.^18^

We fitted multivariable logistic regression models with robust SEs to account for clustering by household. We used a best-subset selection approach, modelling all possible combinations of candidate predictor variables hypothesised to influence an individual's treatment status in round one. The final models consisted of the variables with the lowest Bayesian Information Criterion (appendix p 8).^19^ To estimate the association between non-treatment in round two, three, or four and non-treatment in the previous round, we fitted logistic regression models with robust SEs to account for clustering by household and applied random intercepts for individual. We controlled for the predictors of non-treatment identified for round one as potential confounders. Additionally, we investigated whether a round including school-based deworming or distribution of diethylcarbamazine citrate affected the outcomes for children and adults, respectively.

Multivariable multinomial logistic regression models fitted with robust SEs were also used to account for clustering by household to identify factors associated with part or no treatment versus complete treatment, again assessing all possible subsets of the candidate predictors. For this model, we explored whether excluding rounds in which women of childbearing age (15–49 years) were not treated and were recorded as being pregnant, or having given birth in the previous 2 weeks changed the estimates for all adults. For all models, we examined the effects of different assumptions about individual censoring. For the main analysis we excluded individuals recorded as deceased or migrated, with additional sensitivity analyses to assess various assumptions based on the availability of recorded treatment information (appendix p 3). All dataset assembly and analyses were done in Stata version 15.

### Role of the funding source

The funders had no role in study design, data collection, data analysis, data interpretation, or writing of the report. The corresponding author had full access to all the data in the study and had final responsibility for the decision to submit for publication.

## Results

40 662 individuals in 7834 households surveyed at baseline in the TUMIKIA trial and randomly assigned to receive biannual treatment were considered for inclusion in this analysis. Due to unavailability of treatment information from round two, data from one cluster (310 households and 1748 individuals) were excluded. 25 households were linked to the same household treatment record as another baseline household and were excluded. 89 duplicated individual records were also excluded. Of the remaining 37 458 individuals enumerated in 7499 households and aged 2 years or older at the time of the TUMIKIA trial baseline survey, 1131 (3·0%) were recorded as being deceased or having moved away and, therefore, the final analysis included 36 327 (97·0%) individuals (appendix p 1).

Counts of non-treatment within MDA rounds and overall patterns of non-treatment across the four MDA rounds are shown in the figure. No treatment was recorded for 5310 (32·7%) of 16 236 children and 8260 (41·1%) of 20 091 adults in round one. In children and adults, the occurrence of non-treatment was lowest in round one and increased over time.

**Figure:**
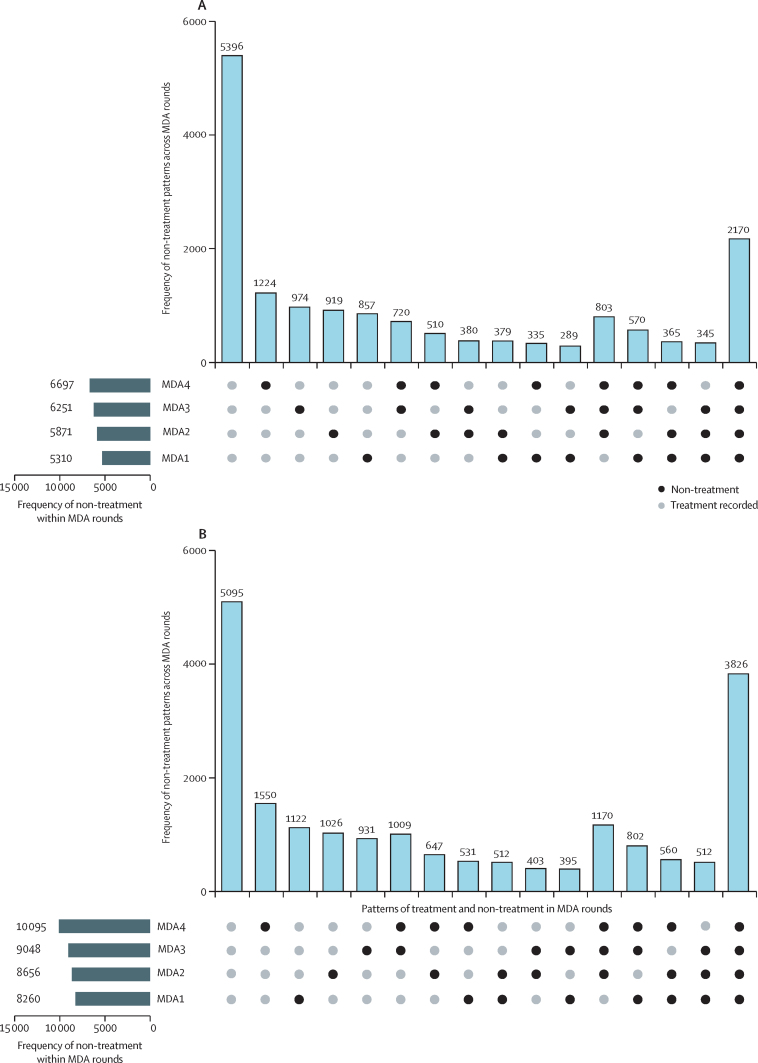
Frequency of non-treatment within MDA rounds and patterns across the four MDA rounds (A) Counts of non-treatment in 16 236 children aged 2–14 years. (B) Counts of non-treatment in 20 091 individuals aged 15 years and older. No treatment during any round is shown on the right, each combination of part treatment in one, two, or three rounds, and complete treatment on the left. MDA=mass drug administration.

Children reported to attend school had lower odds of non-treatment in round one than children reported not to attend school (table 1). The odds of non-treatment were raised substantially by the household head not having been treated, by least poor socioeconomic status, living in a less-remote household, and living in an urban or periurban location. Among adults, age (particularly in the age group 20 to <25 years), sex, and large household size were associated with non-treatment in round one (table 2). Women were less likely than men to have treatment recorded when controlling for age and household size.

**Table 1:** Frequency of non-treatment by individual and household characteristics and association with selected predictors in round one of mass drug administration among children

		**Number (%) of participants (n=16 236)**	**Not treated (%)**	Odds ratio (95% CI)*
**Age group (years)**				
2 to <5		3804 (23·4%)	37·5%	..
5 to <10		6756 (41·6%)	31·6%	..
10 to <15		5676 (35·0%)	30·8%	..
**Sex**				
Male		8283 (51·0%)	32·9%	..
Female		7953 (49·0%)	32·5%	..
**Attends school**				
No		4189 (25·8%)	38·0%	1 (ref)
Yes		12 047 (74·2%)	30·8%	0·66 (0·61–0·72)
**Household**				
Head not treated				
	No	10 817 (66·6%)	22·0%	1 (ref)
	Yes	5419 (33·4%)	54·0%	4·21 (3·77–4·71)
Socioeconomic status				
	Poorest	4813 (29·6%)	31·2%	1 (ref)
	Poor	8463 (52·1%)	32·2%	1·07 (0·94–1·20)
	Least poor	2960 (18·2%)	36·4%	1·32 (1·13–1·55)
Large size				
	No	7820 (48·2%)	32·2%	..
	Yes	8416 (51·8%)	33·2%	..
Remote				
	No	13 095 (80·6%)	33·6%	1 (ref)
	Yes	3141 (19·3%)	28·9%	0·85 (0·73–0·98)
Urban or periurban location				
	No	12 079 (74·4%)	31·9%	1 (ref)
	Yes	4157 (25·6%)	35·0%	1·17 (1·04–1·33)

* Multivariable logistic regression with robust SEs was done to account for household clustering. Odds ratios and 95% CIs are provided for the variables with the lowest Bayesian Information Criterion when all possible subsets of candidate predictors were modelled.

**Table 2:** Frequency of non-treatment by individual and household characteristics and association with selected predictors in round one of mass drug administration among adults

		**Number (%) of participants (n=20 091)**	**Not treated (%)**	Odds ratio (95% CI)*
**Age group (years)**				
15 to <20		3889 (19·4%)	44·8%	1 (ref)
20 to <25		2612 (13·0%)	53·0%	1·39 (1·26–1·54)
25 to <30		2509 (12·5%)	47·4%	1·12 (1·01–1·25)
30 to <35		2243 (11·2%)	41·2%	0·87 (0·78–0·97)
35 to <45		3398 (16·9%)	33·9%	0·64 (0·58–0·70)
45 to <55		2329 (11·6%)	33·2%	0·63 (0·56–0·70)
55 to <65		1682 (8·4%)	32·9%	0·62 (0·55–0·71)
≥65		1429 (7·1%)	37·7%	0·77 (0·68–0·88)
**Sex**				
Male		9364 (46·6%)	39·0%	1 (ref)
Female		10 727 (53·4%)	42·9%	1·14 (1·08–1·20)
**Household**				
Socioeconomic status				
	Poorest	5226 (26·0%)	40·7%	..
	Poor	10 451 (52·0%)	41·1%	..
	Least poor	4414 (22·0%)	41·5%	..
Large size				
	No	12 080 (60·1%)	39·6%	1 (ref)
	Yes	8011 (39·9%)	43·3%	1·13 (1·05–1·22)
Remote				
	No	16 580 (82·5%)	41·1%	..
	Yes	3511 (17·5%)	41·0%	..
Urban or periurban location				
	No	14 174 (70·5%)	40·9%	..
	Yes	5917 (29·5%)	41·5%	..

* Multivariable logistic regression with robust SEs was done to account for household clustering. Odds ratios and 95% CIs are provided for the variables with the lowest Bayesian Information Criterion when all possible subsets of candidate predictors were modelled.

Among children, non-treatment in a previous round was associated with increased odds of non-treatment in the subsequent round (OR 3·60, 95% CI 3·08–4·20) when controlling for school attendance, non-treatment of the household head, household socioeconomic status, household remoteness, and urban or periurban household location. Controlling for whether school-based deworming was implemented during a given round did not change the association (OR 3·59, 95% CI 3·07–4·19). After censoring for lack of treatment register records, household socioeconomic status and remoteness were not selected as predictors of non-treatment, and the association between non-treatment in previous and the subsequent round was attenuated down towards null (appendix p 4).

Adults with no treatment recorded in a given round had substantially increased odds of not having treatment reported in the subsequent round (OR 5·58, 95% CI 5·01–6·21) when controlling for age, sex, and household size. Concurrent distribution of diethylcarbamazine citrate did not meaningfully change the association (OR 5·58, 95% CI 5·02–6·21). When censoring was considered, household size was no longer selected as a predictor of non-treatment and the estimated association between non-treatment in previous and subsequent rounds was attenuated down towards null (appendix p 4).

Across the four rounds of MDA, no treatment was the least likely outcome among children (2170 [13·4%] of 16 236; table 3). Over half received part treatment (8670 [53·4%]), and around a third received complete treatment (5396 [33·2%]). Children who attended school had lower odds of part or no treatment than those who did not attend school. Higher socioeconomic status and living in an urban or periurban location notably increased the odds of receiving part or no treatment. After data censoring, household socioeconomic status was no longer selected as a predictor, but interpretation of the models and other selected predictors did not change meaningfully (appendix p 5).

**Table 3:** Association between baseline individual and household characteristics and part or no treatment in children

		**Complete treatment (n=5396)**	**Part treatment (n=8670)**		**No treatment (n=2170)**	
			Number (%)	Odds ratio (95% CI)*	Number (%)	Odds ratio (95% CI)*
**Age group (years)**						
2 to <5		1214 (31·9%)	1982 (52·1%)	..	608 (16·0%)	..
5 to <10		2327 (34·4%)	3559 (52·7%)	..	870 (12·9%)	..
10 to <15		1855 (32·7%)	3129 (55·1%)	..	692 (12·2%)	..
**Sex**						
Male		2760 (33·3%)	4415 (53·3%)	..	1108 (13·4%)	..
Female		2636 (33·1%)	4255 (53·5%)	..	1062 (13·3%)	..
**Attends school**						
No		1264 (30·2%)	2239 (53·4%)	1 (ref)	686 (16·4%)	1 (ref)
Yes		4132 (34·3%)	6431 (53·4%)	0·86 (0·79–0·93)	1484 (12·3%)	0·63 (0·55–0·71)
**Household**						
Socioeconomic status						
	Poorest	1631 (33·9%)	2619 (54·4%)	1 (ref)	563 (11·7%)	1 (ref)
	Poor	2878 (34·0%)	4453 (52·6%)	0·96 (0·85–1·09)	1132 (13·4%)	1·17 (0·99–1·39)
	Least poor	887 (30·0%)	1598 (54·0%)	1·15 (0·98–1·34)	475 (16·0%)	1·67 (1·33–2·08)
Large size						
	No	2552 (32·6%)	4235 (54·2%)	..	1033 (13·2%)	..
	Yes	2844 (33·8%)	4435 (52·7%)	..	1137 (13·5%)	..
Remote						
	No	4302 (32·8%)	6981 (53·3%)	..	1812 (13·8%)	..
	Yes	1094 (34·8%)	1689 (53·8%)	..	358 (11·4%)	..
Urban or periurban location						
	No	4156 (34·4%)	6317 (52·3%)	1 (ref)	1606 (13·3%)	1 (ref)
	Yes	1240 (29·8%)	2353 (56·6%)	1·27 (1·12–1·44)	564 (13·6%)	1·22 (1·03–1·45)

* Multivariable logistic regression with robust SEs was done to account for household clustering. Odds ratios and 95% CIs are provided for the variables with the lowest Bayesian Information Criterion when all possible subsets of candidate predictors were modelled.

More than half of adults received part treatment (11 170 [55·6%] of 20 091), no treatment was received by 3826 (19·0%), and a quarter received complete treatment (5095 [25·4%]; table 4). The age group from 20 to younger than 25 years had the highest odds of receiving part or no treatment when controlling for sex and household size, whereas odds were reduced in those aged 30 years and older. Women had higher odds of receiving part or no treatment than men when controlling for age and household size. With censoring, only age and sex were selected as predictors of non-treatment (appendix p 6). In these alternate models, women had higher odds of receiving part treatment but lower odds of no treatment than men, and adults aged 20–35 years had the highest odds of receiving part or no treatment.

**Table 4:** Association between baseline individual and household characteristics and part or no treatment in adults

		**Complete treatment (n=5095)**	**Part treatment (n=11 170)**		**No treatment (n=3826)**	
			Number (%)	Odds ratio (95% CI)*	Number (%)	Odds ratio (95% CI)*
**Age group (years)**						
15 to <20		698 (17·9%)	2318 (59·6%)	1 (ref)	873 (22·4%)	1 (ref)
20 to <25		329 (12·6%)	1555 (59·5%)	1·41 (1·22–1·63)	728 (27·9%)	1·81 (1·53–2·14)
25 to <30		464 (18·5%)	1474 (58·7%)	0·94 (0·82–1·08)	571 (22·8%)	1·02 (0·86–1·20)
30 to <35		589 (26·3%)	1276 (56·9%)	0·65 (0·57–0·74)	378 (16·8%)	0·53 (0·45–0·62)
35 to <45		1088 (32·0%)	1853 (54·5%)	0·51 (0·46–0·57)	457 (13·4%)	0·35 (0·30–0·40)
45 to <55		830 (35·6%)	1185 (50·9%)	0·43 (0·38–0·49)	314 (13·5%)	0·31 (0·27–0·37)
55 to <65		628 (37·3%)	802 (47·7%)	0·39 (0·34–0·45)	252 (15·0%)	0·34 (0·28–0·40)
≥65		469 (32·8%)	707 (49·5%)	0·46 (0·39–0·53)	253 (17·7%)	0·46 (0·38–0·55)
**Sex**						
Male		2554 (27·3%)	5026 (53·7%)	1 (ref)	1784 (19·0%)	1 (ref)
Female		2541 (23·7%)	6144 (57·3%)	1·18 (1·11–1·25)	2042 (19·0%)	1·09 (1·01–1·18)
**Household**						
Socioeconomic status						
	Poorest	1321 (25·3%)	2976 (56·9%)	..	929 (17·8%)	..
	Poor	2637 (25·2%)	5828 (55·8%)	..	1986 (19·0%)	..
	Least poor	1137 (25·8%)	2366 (53·6%)	..	911 (20·6%)	..
Large size						
	No	3202 (26·5%)	6749 (55·9%)	1 (ref)	2129 (17·6%)	1 (ref)
	Yes	1893 (23·6%)	4421 (55·2%)	1·03 (0·94–1·12)	1697 (21·2%)	1·24 (1·10–1·39)
Remote						
	No	4206 (25·4%)	9167 (55·3%)	..	3207 (19·3%)	..
	Yes	889 (25·3%)	2003 (57·0%)	..	619 (17·6%)	..
Urban or periurban location						
	No	3678 (25·9%)	7802 (55·0%)	..	2694 (19·0%)	..
	Yes	1417 (23·9%)	3368 (56·9%)	..	1132 (19·1%)	..

* Multivariable logistic regression with robust SEs was done to account for household clustering. Odds ratios and 95% CIs are provided for the variables with the lowest Bayesian Information Criterion when all possible subsets of candidate predictors were modelled.

Among 20 091 adults, 8694 women (43·3%) were of childbearing age and 1421 (16·3%) of these had pregnancy or birth within the previous 2 weeks recorded as the reason for non-treatment during at least one of the MDA rounds. 274 (19·3%) of these 1421 women did not receive treatment in two or more rounds. After excluding the rounds for women who were ineligible because of pregnancy or a recent birth, selected predictors were sex, age, large household size, and urban or periurban location, and women had lower odds than men of receiving part or no treatment (appendix p 7).

## Discussion

Despite recognition of the potential threat of systematic non-treatment on the effectiveness of repeated MDA,^9^, ^11^, ^20^ few longitudinal studies have assessed treatment at the individual level in large-scale MDA programmes. By combining household surveys and longitudinal treatment data, we were able to assess the number of treatments received by individuals across four rounds of MDA over 2 years, in which drugs were delivered door-to-door by community health volunteers and in schools. Individuals not treated in a given round were more likely to miss treatment in later rounds, irrespective of the sociodemographic factors that affect reach and uptake overall. We found the greatest odds of non-treatment in those aged 15–30 years. This age group has previously been shown to harbour the highest rate of hookworm infections in Kwale county.^13^ The substantial degree of non-treatment in this group could have important implications for the feasibility of achieving sustained morbidity or transmission control.

In children, the factor most strongly associated with receipt of treatment in the first MDA round was the treatment status of the household head, and this factor was predictive independent of school attendance and household socioeconomic status and location. Receipt of treatment during MDA for trachoma, soil-transmitted helminths, schistosomiasis, and onchocerciasis has similarly been associated with treatment of the household head and is found to cluster within households.^18^, ^21^, ^22^, ^23^ Household clustering highlights the importance of adequate community engagement and sensitisation. As previously shown in the TUMIKIA trial and other studies, community engagement during MDA programmes can address perceptions about safety, effectiveness, and need for repeated treatment, and build trust and the demand for treatment.^6^, ^7^, ^8^, ^15^, ^24^

We found that women were more likely than men to receive part or no treatment with adjustment for age and household size. However, this pattern was reversed when women ineligible for treatment due to pregnancy or birth within the previous 2 weeks were excluded. Previous studies have noted lower treatment rates among women during MDA for lymphatic filariasis and onchocerciasis, which were attributed to ineligibility due to pregnancy and related safety concerns.^7^, ^20^ WHO conditionally recommends that women of reproductive age and pregnant women in the second or third trimester be included in deworming programmes.^10^ Our results suggest that community-wide MDA can be an effective platform to reach this group, but in practice, additional strategies may be needed to ensure high coverage.^25^

Improved data collection and management solutions to identify, locate, and treat individuals within and across MDA rounds will be needed to reduce systematic non-treatment. Prepopulating paper registers with individual information can facilitate targeting of individuals who were absent from the household during MDA, although this approach requires considerable logistical input. Additionally, it would still not ensure treatment of temporarily absent individuals, which was the most common recorded reason for non-treatment among adults in this analysis (appendix p 2). The use of mobile health approaches, such as SMS monitoring,^26^ District Health Information Software 2^27^ and similar national systems,^28^ or smartphone-based electronic treatment registers^29^ to support data collection and decision making has been included in WHO guidelines.^30^ Although such interventions need to be further assessed,^31^ digital tracking approaches could improve targeting and treatment of urban and migrant populations, reduce costs, and increase coverage through real-time monitoring, and would in turn reduce systematic non-treatment.

Our analysis is subject to several limitations. The deworming activities were done by the Kwale county government, but financial, human, and in-kind resources were provided by the trial infrastructure. Whether similarly high coverage could be achieved during normal programmatic activities is unclear and might limit the generalisability of our results. Although our use of treatment register data eliminated recall bias and was representative of programmatic conditions, the lack of an individual census is an important limitation. We employed data officers for data abstraction and record linkage who were familiar with local languages and naming conventions, but errors might still have occurred when linking individual data longitudinally. Similarly, absence of data for an individual in a round might be due to incomplete data, absence during treatment, or permanent migration away from the study area. Towards addressing these limitations, alongside the main analysis that assumed a fixed cohort, we presented results of two approaches for censoring individuals without a treatment record during a specific and subsequent rounds.

In conclusion, we identified demographic subgroups in which the likelihood of not having any treatment recorded during multiple MDA rounds was increased, and found that non-treatment was associated across rounds. Modelling studies have suggested that soil-transmitted helminth infections in endemic communities become increasingly clustered as disease prevalence drops, determined in part by patterns of individual participation during MDA, with important consequences for the probability of achieving transmission interruption.^32^, ^33^ Developing a stronger empirical understanding of the effects of systematic non-treatment on transmission of soil-transmitted helminths and other neglected tropical diseases, and the identification of new approaches to ensure treatment of identified subgroups, remain urgent priorities.

For **LSHTM Data Compass** see https://datacompass.lshtm.ac.uk/1311/

For **World-Pop estimates of population density** see http://www.worldpop.org.uk

For **Open Street Map data** see https://www.openstreetmap.org

## Data sharing

De-identified individual participant data will be made available on publication to members of the scientific and medical communities for non-commercial use only. Requests may be made via email to the corresponding author. Data will be stored in Data Compass, the electronic respository of the London School of Hygiene & Tropical Medicine. The data collection instruments used in the TUMIKIA trial are available for download from LSHTM Data Compass.
