# Supplementary material for: Patterns of individual non-treatment during multiple rounds of mass drug administration for control of soil-transmitted helminths in the TUMIKIA trial, Kenya: a secondary longitudinal analysis
Source: Lancet Glob Health. 2020 Oct 15;8(11):e1418–26. doi: 10.1016/S2214-109X(20)30344-2 (PMC7564382; doi:10.1016/S2214-109X(20)30344-2)
Supplement: Supplementary appendix [file mmc1.pdf]

THE LANCET
Global Health

Supplementary appendix

This appendix formed part of the original submission and has been peer reviewed. We post it as supplied by the authors.

Supplement to: Oswald WE, Kepha S, Halliday KE, et al. Patterns of individual non-treatment during multiple rounds of mass drug administration for control of soil-transmitted helminths in the TUMIKIA trial, Kenya: a secondary longitudinal analysis. *Lancet Glob Health* 2020; **8:** e1418–26.

Supplementary Information

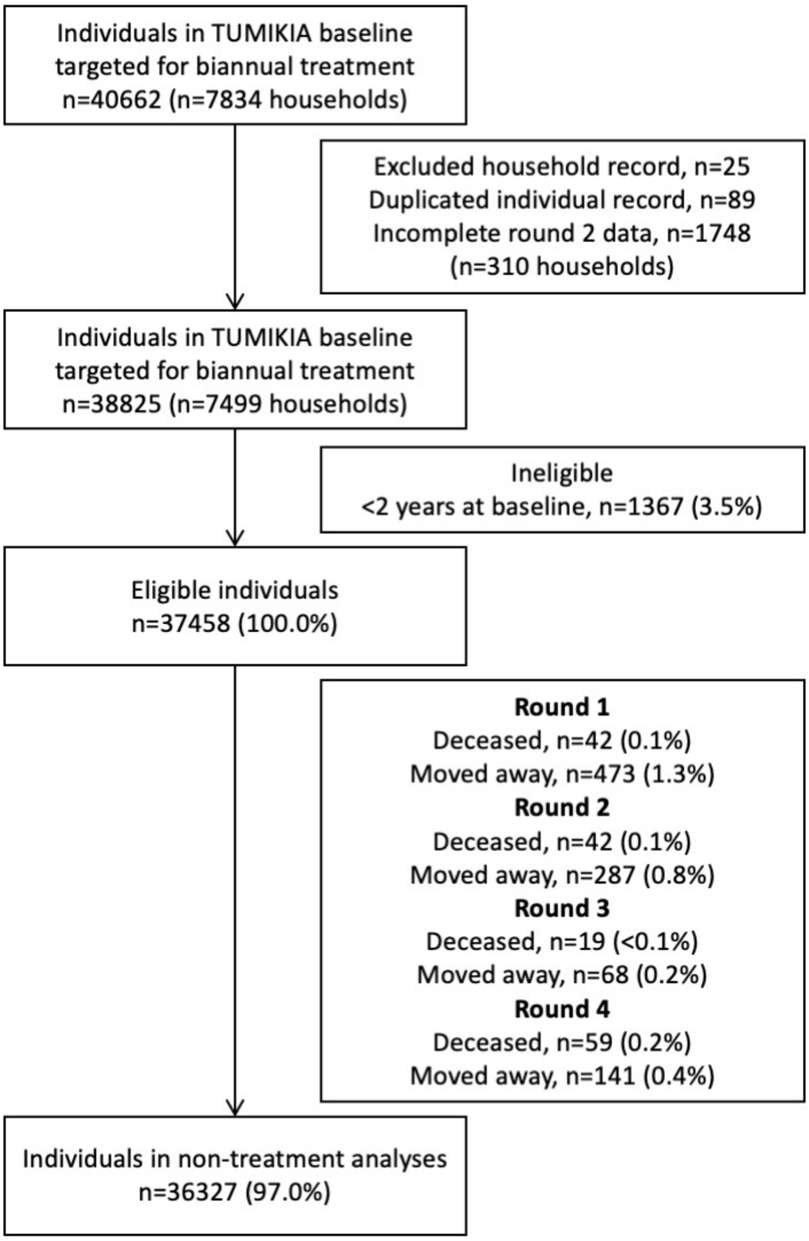

Figure S1 – Flow chart of participants enumerated in the TUMIKIA baseline survey who were included in current analyses. Eligible individuals included those age 2 years or older during the baseline survey not recorded as deceased or moved away during a treatment round. The proportion of eligible individuals included in the analysis was 97.0% (36327/37458).

Reasons for Non-treatment

During MDA, community health volunteers recorded the reason for non-treatment in paper registers using a pre-coded list of options. Subsequently during digitisation, if no reason was recorded in the register this was digitised as a 'missing response'. For our analyses, we used the individual's age as recorded during the baseline household questionnaire, which did not exactly correspond in some cases to the age recorded in the treatment registers.

Table S1 – Frequency of recorded reasons for non-treatment among 24129 children aged 2 to 14 with no treatment recorded by MDA round, Kenya, 2015-2016

	MDA 1		MDA 2		MDA 3		MDA 4		Total	
	no.	%	no.	%	no.	%	no.	%	no.	%
Temporarily absent	68	1.28	177	3.01	57	0.91	205	3.06	507	2.10
Pregnant	2	0.04	2	0.03	4	0.06	7	0.10	15	0.06
Recent birth	4	0.08	3	0.05	4	0.06	0	0.00	11	0.05
Refused	23	0.43	126	2.15	34	0.54	111	1.66	294	1.22
Under 2 years	159	2.99	103	1.75	64	1.02	37	0.55	363	1.50
Spat tablet out	6	0.11	5	0.09	3	0.05	1	0.01	15	0.06
Sick	40	0.75	39	0.66	20	0.32	41	0.61	140	0.58
Other	9	0.17	26	0.44	14	0.22	20	0.30	69	0.29
Missing response*	247	4.65	110	1.87	--	--	64	0.96	421	1.74
No record	4,752	89.49	5,280	89.93	6051	96.80	6211	92.74	22294	92.40

* During data assembly for MDA 3, missing responses were coded as missing data, so these observations are included here as 'no record.'

Table S2 – Frequency of recorded reasons for non-treatment among 36059 individuals aged 15 years and older with no treatment recorded by MDA round, Kenya, 2015-2016

	MDA 1		MDA 2		MDA 3		MDA 4		Total	
	no.	%	no.	%	no.	%	no.	%	no.	%
Temporarily absent	638	7.72	705	8.14	602	6.65	764	7.57	2709	7.51
Pregnant	373	4.52	381	4.40	301	3.33	288	2.85	1343	3.72
Recent birth	280	3.39	49	0.57	36	0.40	41	0.41	406	1.13
Refused	219	2.65	353	4.08	320	3.54	396	3.92	1288	3.57
Under 2 years	11	0.13	10	0.12	10	0.11	8	0.08	39	0.11
Spat tablet out	6	0.07	3	0.03	0	0.00	2	0.02	11	0.03
Sick	238	2.88	136	1.57	181	2.00	203	2.01	758	2.10
Other	64	0.77	73	0.84	158	1.75	129	1.28	424	1.18
Missing response*	150	1.82	145	1.68	--	--	121	1.20	416	1.15
No record	6281	76.04	6801	78.57	7440	82.23	8143	80.66	28665	79.49

* During data assembly for MDA 3, missing responses were coded as missing data, so these observations are included here as 'no record.'

Data Censoring

For the main analysis we excluded only individuals recorded in the treatment data as either deceased or migrated in any round. We conducted additional analyses to examine the impact of different assumptions about censoring of individuals based on the availability of recorded treatment information. Prior to generating the non-treatment indicator, we flagged individuals with no linked treatment information for a given round and no linked information for any subsequent rounds. These flags were used to exclude these individuals from analyses to test the assumptions. It is important to clarify that if an individual had no recorded treatment information in a given round but had recorded information from a later round, they were classed as not treated for the earlier round and not censored. The numbers of individuals included in the main and alternate analyses or censored along with frequency of non-treatment are presented in Table S3.

All individuals with recorded information from at least one round were included in the analysis to identify predictors of non-treatment in round one (Table S4). Then, for the estimation of the association of non-treatment with subsequent non-treatment, we censored individuals if they had no treatment register record during rounds two or three and following rounds and no record at round four (Sensitivity 1). However, because there was no subsequent round after round four, this assumption meant all individuals without a record at round four were excluded (rather than being classed as 'not treated'), so we also examined the impact of ignoring this censoring at round four (Sensitivity 2). This latter approach would not affect the selection of round one predictors, only the estimation of the association of non-treatment between rounds.

We also examined the impact of these assumptions on the analysis of factors associated with overall non-treatment by excluding rounds where the individual was considered to be censored from the determination of complete, partial, or no treatment. The results of the alternate analyses are presented below (Tables S5 – S8).

Finally, as described in the main text, we examined the impact of excluding rounds where women of child-bearing age were recorded to be ineligible for treatment because of recorded pregnancy, breastfeeding, or having recently given birth. The results of this analysis are presented below (Table S9).

Table S3 - Numbers of individuals included in different analyses and frequency of non-treatment by round and age ranges

	Included in	2 – 14 years			15+ years			All		
Round	Analyses	no.	NT	%	no.	NT	%	no.	NT	%
1	Main	16236	5310	32·7	20091	8260	41·1	36327	13570	37·4
	Sensitivity 1 & 2	14234	3308	23·2	17009	5178	30·4	31243	8486	27·2
	Censored	2002	··	··	3082	··	··	5084	··	··
2	Main	16236	5871	36·2	20091	8656	43·1	36327	14527	40·0
	Sensitivity 1 & 2	13505	3140	23·2	16039	4604	28·7	29544	7744	26·2
	Censored	729	··	··	970	··	··	1699	··	··
3	Main	16236	6251	38·5	20091	9048	45·0	36327	15299	42·1
	Sensitivity 1 & 2	12235	2250	18·4	14538	3495	24·0	26773	5745	21·5
	Censored	1270	··	··	1501	··	··	2771	··	··
4	Main	16236	6697	41·2	20091	10095	50·2	36327	16792	46·2
	Sensitivity 1	10028	489	4·9	11950	1954	16·3	21978	2443	11·1
	Censored	2207	··	··	2588	··	··	4795	··	··
	Sensitivity 2	12235	2696	22·0	14538	4542	31·2	26773	7238	27·0
	Censored	0	··	··	0	··	··	0	··	··

Predictors of non-treatment during round one and association of non-treatment with subsequent non-treatment

Table S4 – Frequency of non-treatment by individual and household characteristics and association of selected predictors with non-treatment during round one of mass drug administration among 14234 children aged 2 to 14 years and 17009 individuals aged 15 years and older in Kwale County, Kenya, 2015

	2 – 14 years					15+ years				
	n	%	NT (%)	OR†	95% CI	n	%	NT (%)	OR†	95% CI
**Age, years**										
2-<5	3267	22·9	27·2	··	··	··	··	··	··	··
5-<10	5939	41·7	22·2	··	··	··	··	··	··	··
10-<15	5028	35·3	21·9	··	··	··	··	··	··	··
15-<20	··	··	··	··	··	3134	18·4	31·5	1·00	··
20-<25	··	··	··	··	··	2002	11·8	38·6	1·35	(1·20, 1·52)
25-<30	··	··	··	··	··	2036	12·0	35·2	1·16	(1·02, 1·31)
30-<35	··	··	··	··	··	1970	11·6	33·1	1·06	(0·94, 1·20)
35-<45	··	··	··	··	··	3061	18·0	26·6	0·78	(0·70, 0·87)
45-<55	··	··	··	··	··	2102	12·4	26·0	0·76	(0·68, 0·86)
55-<65	··	··	··	··	··	1487	8·7	24·1	0·69	(0·60, 0·80)
65+	··	··	··	··	··	1217	7·2	26·9	0·80	(0·69, 0·93)
**Sex**										
Male	7265	51·0	23·5	··	··	7994	47·0	28·6	1·00	··
Female	6969	49·0	23·0	··	··	9015	53·0	32·1	1·14	(1·07, 1·21)
**Attends School**										
No	3595	25·3	27·8	1·00	··	··	··	··	··	··
Yes	10639	74·7	21·7	0·67	(0·61, 0·74)	··	··	··	··	··
**Household Head NT**										
No	9794	68·8	13·9	1·00	··	··	··	··	··	··
Yes	4440	31·2	43·9	4·93	(4·35, 5·60)	··	··	··	··	··
**Household SES**										
Poorest	4300	30·2	23·0	··	··	4484	26·4	30·9	··	··
Poor	7416	52·1	22·6	··	··	8870	52·1	30·6	··	··
Least Poor	2518	17·7	25·3	··	··	3655	21·5	29·4	··	··
**Large Household**										
No	6880	48·3	22·9	··	··	10412	61·2	30·0	··	··
Yes	7354	51·7	23·5	··	··	6597	38·8	31·2	··	··
**Remote Household**										
No	11419	80·2	23·9	··	··	13988	82·2	30·2	··	··
Yes	2815	19·8	20·6	··	··	3021	17·8	31·4	··	··
**Urban Household**										
No	10598	74·5	22·4	1·00	··	12040	70·8	30·5	··	··
Yes	3636	25·5	25·7	1·27	(1·11, 1·46)	4969	29·2	30·3	··	··

†Multivariable logistic regression with robust errors for household clustering with predictors selected from all possible subsets of candidate predictors using Bayesian Information Criterion (BIC). Acronyms: NT=Non-Treatment; OR=Odds Ratio; CI=Confidence Interval; SES=Socioeconomic Status

Among 13505 children not censored at round two, previous non-treatment was associated with increased odds of non-treatment at a subsequent round (OR 2·72, 95%CI 1·79, 2·88), controlling for school attendance, head of household non-treatment, and urban household. Controlling for the same factors, but ignoring censoring at round four, among the same 13505 children, previous non-treatment was more weakly associated with increased odds of non-treatment at a subsequent round (OR 1·16, 95%CI 0·99, 1·34).

Among 16039 adults not censored at round two, those without recorded treatment during the previous round were 2·6 times more likely to not have treatment reported during the subsequent round compared to those with treatment recorded (OR 2·58, 95%CI 2·34, 2·85), controlling for age and sex. Ignoring censoring at round four, among the same adults, previous non-treatment was more weakly associated with increased odds of non-treatment at a subsequent round (OR 1·80, 95%CI 1·63, 1·98), controlling for age and sex.

Frequency of overall non-treatment and associated factors

Table S5 – Association of baseline individual and household characteristics with partial or no treatment (relative to complete treatment) among 14234 children aged 2 to 14 years in Kwale County, Kenya during uncensored biannual mass drug administration rounds (Sensitivity 1), 2015-2016

	Complete treatment		Partial treatment				No treatment			
**Characteristic**	**n**	**%**	**n**	**%**	**OR†**	**95% CI**	**n**	**%**	**OR†**	**95% CI**
**Age, years**										
2-<5	1718	52·6	1478	45·2	··	··	71	2·2	··	··
5-<10	3296	55·5	2590	43·6	··	··	53	0·9	··	··
10-<15	2712	53·9	2272	45·2	··	··	44	0·9	··	··
**Sex**										
Male	3956	54·4	3219	44·3	··	··	90	1·2	··	··
Female	3770	54·1	3121	44·8	··	··	78	1·1	··	··
**Attends school**										
No	1860	51·7	1643	45·7	1·00	··	92	2·6	1·00	··
Yes	5866	55·1	4697	44·1	0·91	(0·83, 0·98)	76	0·7	0·26	(0·19, 0·36)
**Household SES**										
Poorest	2368	55·1	1882	43·8	··	··	50	1·2	··	··
Poor	4027	54·3	3304	44·5	··	··	85	1·1	··	··
Least Poor	1331	52·9	1154	45·8	··	··	33	1·3	··	··
**Large Household**										
No	3759	54·6	3028	44·0	··	··	93	1·3	··	··
Yes	3967	53·9	3312	45·0	··	··	75	1·0	··	··
**Remote Household**										
No	6149	53·8	5134	45·0	··	··	136	1·2	··	··
Yes	1577	56·0	1206	42·8	··	··	32	1·1	··	··
**Urban Household**										
No	5818	54·9	4655	43·9	··	··	125	1·2	··	··
Yes	1908	52·5	1689	46·3	··	··	43	1·2	··	··

†Multinomial logistic regression with robust errors for household clustering selected from all possible subsets of candidate predictors using Bayesian Information Criterion (BIC). Acronyms: OR=Odds Ratio; CI=Confidence Interval; SES=Socioeconomic Status

Table S6 – Association of baseline individual and household characteristics with partial or no treatment (relative to complete treatment) among 14234 children aged 2 to 14 years in Kwale County, Kenya during uncensored biannual mass drug administration rounds (Sensitivity 2, including individuals with no record in round four), 2015-2016

	Complete treatment		Partial treatment				No treatment			
**Characteristic**	**n**	**%**	**n**	**%**	**OR†**	**95% CI**	**n**	**%**	**OR†**	**95% CI**
**Age, years**										
2-<5	1506	46·1	1690	51·7	··	··	71	2·2	··	··
5-<10	2900	48·8	2986	50·3	··	··	53	0·9	··	··
10-<15	2305	45·8	2679	53·3	··	··	44	0·9	··	··
**Sex**										
Male	3413	47·0	3762	51·8	··	··	90	1·2	··	··
Female	3298	47·3	3593	51·6	··	··	78	1·1	··	··
**Attends school**										
No	1612	44·8	1891	52·6	1·00	··	92	2·6	1·00	··
Yes	5099	47·9	5464	51·4	0·90	(0·83, 0·98)	76	0·7	0·26	(0·19, 0·36)
**Household SES**										
Poorest	2048	47·6	2202	51·2	··	··	50	1·2	··	··
Poor	3528	47·6	3803	51·3	··	··	85	1·1	··	··
Least Poor	1135	45·1	1350	53·6	··	··	33	1·3	··	··
**Large Household**										
No	3235	47·0	3552	51·6	··	··	93	1·3	··	··
Yes	3476	47·3	3803	51·7	··	··	75	1·0	··	··
**Remote Household**										
No	5357	46·9	5926	51·9	··	··	136	1·2	··	··
Yes	1354	48·1	1429	50·8	··	··	32	1·1	··	··
**Urban Household**										
No	5123	48·3	5350	50·5	1·00	··	125	1·2	1·00	··
Yes	1588	43·7	2005	55·1	1·22	(1·08, 1·36)	43	1·2	1·23	(0·85, 1·79)

†Multinomial logistic regression with robust errors for household clustering selected from all possible subsets of candidate predictors using Bayesian Information Criterion (BIC). Acronyms: OR=Odds Ratio; CI=Confidence Interval; SES=Socioeconomic Status

Table S7 – Association of baseline individual and household characteristics with partial or no treatment (relative to complete treatment) during uncensored biannual mass drug administration rounds (Sensitivity 1) among 17009 individuals aged 15+ years in Kwale County, Kenya, 2015-2016

	Complete treatment		Partial treatment				No treatment			
Characteristic	n	%	n	%	OR†	95% CI	n	%	OR†	95% CI
**Age, years**										
15-<20	1346	43·0	1670	53·3	1·00	··	118	3·8	1·00	··
20-<25	695	34·7	1189	59·4	1·36	(1·21, 1·54)	118	5·9	2·01	(1·53, 2·65)
25-<30	783	38·5	1155	56·7	1·18	(1·04, 1·33)	98	4·8	1·51	(1·13, 2·02)
30-<35	800	40·6	1065	54·1	1·06	(0·95, 1·20)	105	5·3	1·55	(1·17, 2·06)
35-<45	1400	45·7	1541	50·3	0·88	(0·80, 0·98)	120	3·9	0·99	(0·76, 1·29)
45-<55	1043	49·6	972	46·2	0·75	(0·67, 0·84)	87	4·1	0·95	(0·71, 1·27)
55-<65	779	52·4	651	43·8	0·67	(0·59, 0·77)	57	3·8	0·83	(0·60, 1·16)
65+	598	49·1	578	47·5	0·78	(0·68, 0·90)	41	3·4	0·77	(0·52, 1·13)
**Sex**										
Male	3581	44·8	3999	50·0	1·00	··	414	5·2	1·00	··
Female	3863	42·8	4822	53·5	1·08	(1·01, 1·14)	330	3·7	0·69	(0·60, 0·80)
**Household SES**										
Poorest	1900	42·4	2397	53·5	··	··	187	4·2	··	··
Poor	3826	43·1	4639	52·3	··	··	405	4·6	··	··
Least Poor	1718	47·0	1785	48·8	··	··	152	4·2	··	··
**Large Household**										
No	4568	43·9	5383	51·7	··	··	461	4·4	··	··
Yes	2876	43·6	3438	52·1	··	··	283	4·3	··	··
**Remote Household**										
No	6167	44·1	7206	51·5	··	··	615	4·4	··	··
Yes	1277	42·3	1615	53·5	··	··	129	4·3	··	··
**Urban Household**										
No	5202	43·2	6278	52·1	··	··	560	4·6	··	··
Yes	2242	45·1	2543	51·2	··	··	184	3·7	··	··

†Multinomial logistic regression with robust errors for household clustering selected from all possible subsets of candidate predictors using Bayesian Information Criterion (BIC). Acronyms: OR=Odds Ratio; CI=Confidence Interval; SES=Socioeconomic Status

Table S8 – Association of baseline individual and household characteristics with partial or no treatment (relative to complete treatment) during uncensored biannual mass drug administration rounds (Sensitivity 2, including individuals with no record in round four) among 17009 individuals aged 15+ years in Kwale County, Kenya, 2015-2016

	Complete treatment		Partial treatment				No treatment			
Characteristic	n	%	n	%	OR†	95% CI	n	%	OR†	95% CI
**Age, years**										
15-<20	1124	35·9	1892	60·4	1·00	··	118	3·8	1·00	··
20-<25	570	28·5	1314	65·6	1·36	(1·20, 1·54)	118	5·9	2·05	(1·55, 2·70)
25-<30	674	33·1	1264	62·1	1·10	(0·97, 1·24)	98	4·8	1·46	(1·09, 1·96)
30-<35	705	35·8	1160	58·9	0·97	(0·86, 1·09)	105	5·3	1·47	(1·10, 1·96)
35-<45	1233	40·3	1708	55·8	0·82	(0·74, 0·91)	120	3·9	0·94	(0·72, 1·23)
45-<55	943	44·9	1072	51·0	0·67	(0·60, 0·75)	87	4·1	0·88	(0·66, 1·18)
55-<65	702	47·2	728	49·0	0·62	(0·54, 0·70)	57	3·8	0·77	(0·55, 1·08)
65+	547	44·9	629	51·7	0·68	(0·59, 0·79)	41	3·4	0·70	(0·48, 1·04)
**Sex**										
Male	3146	39·3	4434	55·5	1·00	··	414	5·2	1·00	··
Female	3352	37·2	5333	59·2	1·08	(1·02, 1·15)	330	3·7	0·70	(0·61, 0·81)
**Household SES**										
Poorest	1678	37·4	2619	58·4	··	··	187	4·2	··	··
Poor	3335	37·6	5130	57·8	··	··	405	4·6	··	··
Least Poor	1485	40·6	2018	55·2	··	··	152	4·2	··	··
**Large Household**										
No	4013	38·5	5938	57·0	··	··	461	4·4	··	··
Yes	2485	37·7	3829	58·0	··	··	283	4·3	··	··
**Remote Household**										
No	5386	38·5	7987	57·1	··	··	615	4·4	··	··
Yes	1112	36·8	1780	58·9	··	··	129	4·3	··	··
**Urban Household**										
No	4611	38·3	6869	57·0	··	··	560	4·6	··	··
Yes	1887	38·0	2898	58·3	··	··	184	3·7	··	··

†Multinomial logistic regression with robust errors for household clustering selected from all possible subsets of candidate predictors using Bayesian Information Criterion (BIC). Acronyms: OR=Odds Ratio; CI=Confidence Interval; SES=Socioeconomic Status

Table S9 – Association of baseline individual and household characteristics with partial or no treatment (relative to complete treatment) during two or more rounds of biannual mass drug administration among 20087 individuals aged 15+ years, excluding rounds for women ineligible for reported pregnancy, breastfeeding, or a recent birth, in Kwale County, Kenya, 2015-2016

	Complete treatment		Partial treatment				No treatment			
**Characteristic**	**n**	**%**	**n**	**%**	**OR†**	**95% CI**	**n**	**%**	**OR†**	**95% CI**
**Age, years**										
15-<20	744	19·1	2272	58·4	1·00	··	873	22·4	1·00	··
20-<25	485	18·6	1399	53·6	0·96	(0·84, 1·10)	728	27·9	1·33	(1·14, 1·56)
25-<30	685	27·3	1253	50·0	0·62	(0·55, 0·71)	567	22·6	0·75	(0·65, 0·88)
30-<35	742	33·1	1123	50·1	0·51	(0·45, 0·58)	378	16·8	0·46	(0·39, 0·54)
35-<45	1203	35·4	1738	51·1	0·48	(0·43, 0·54)	457	13·4	0·34	(0·29, 0·39)
45-<55	836	35·9	1179	50·6	0·47	(0·41, 0·53)	314	13·5	0·33	(0·28, 0·39)
55-<65	628	37·3	802	47·7	0·42	(0·37, 0·49)	252	15·0	0·36	(0·30, 0·43)
65+	469	32·8	707	49·5	0·50	(0·43, 0·58)	253	17·7	0·49	(0·40, 0·59)
**Sex**										
Male	2554	27·3	5026	53·7	1·00	··	1784	19·0	1·00	··
Female	3238	30·2	5447	50·8	0·84	(0·79, 0·89)	2038	19·0	0·86	(0·80, 0·93)
**Household SES**										
Poorest	1521	29·1	2776	53·1	··	··	927	17·7	··	··
Poor	3019	28·9	5446	52·1	··	··	1984	19·0	··	··
Least Poor	1252	28·4	2251	51·0	··	··	911	20·6	··	··
**Large Household**										
No	3665	30·3	6286	52·0	1·00	··	2125	17·6	1·00	··
Yes	2127	26·5	4187	52·3	1·06	(0·97, 1·15)	1697	21·2	1·26	(1·13, 1·41)
**Remote Household**										
No	4724	28·5	8649	52·2	··	··	3203	19·3	··	··
Yes	1068	30·4	1824	51·9	··	··	619	17·6	··	··
**Urban Household**										
No	4233	29·9	7247	51·1	1·00	··	2691	19·0	1·00	··
Yes	1559	26·3	3226	54·5	1·19	(1·09, 1·31)	1131	19·1	1·12	(0·99, 1·26)

†Multinomial logistic regression with robust errors for household clustering selected from all possible subsets of candidate predictors using Bayesian Information Criterion (BIC). Acronyms: OR=Odds Ratio; CI=Confidence Interval; SES=Socioeconomic Status

Multiple testing

To select predictors of non-treatment during round one, we fit all possible subsets and selected those predictors in the model with the lowest Bayesian Information Criteria. For a sample size of 100, optimisation of the BIC corresponds to significance-based selection (i.e. comparison of two hierarchically nested models with a difference of one DF) at a significance level of 0.032.[1] This value is calculated by:

$$\alpha_{BIC}(DF, n) = 1 - F_{\chi 2, DF}(\log(n) \cdot DF)$$

As an example, our model selection process included data for 16236 children, and we calculate that this corresponds to significance-based selection at a significance level of 0.002.

1. Heinze G, Wallisch C, Dunkler D. Variable selection - A review and recommendations for the practicing statistician. *Biom J* 2018; **60**(3): 431-49.
